# PCA-based bootstrap confidence interval tests for gene-disease association involving multiple SNPs

**DOI:** 10.1186/1471-2156-11-6

**Published:** 2010-01-26

**Authors:** Qianqian Peng, Jinghua Zhao, Fuzhong Xue

**Affiliations:** 1 Department of Epidemiology and Health Statistics, School of Public Health, Shandong University, Jinan 250012, PR China; 2 MRC Epidemiology Unit, Institute of Metabolic Science, Addenbrooke's Hospital, Cambridge CB2 0QQ, UK

## Abstract

**Background:**

Genetic association study is currently the primary vehicle for identification and characterization of disease-predisposing variant(s) which usually involves multiple single-nucleotide polymorphisms (SNPs) available. However, SNP-wise association tests raise concerns over multiple testing. Haplotype-based methods have the advantage of being able to account for correlations between neighbouring SNPs, yet assuming Hardy-Weinberg equilibrium (*HWE*) and potentially large number degrees of freedom can harm its statistical power and robustness. Approaches based on principal component analysis (*PCA*) are preferable in this regard but their performance varies with methods of extracting principal components (*PC*s).

**Results:**

*PCA*-based bootstrap confidence interval test (*PCA-BCIT*), which directly uses the *PC *scores to assess gene-disease association, was developed and evaluated for three ways of extracting *PC*s, i.e., cases only(*CAES*), controls only(*COES*) and cases and controls combined(*CES*). Extraction of *PC*s with *COES *is preferred to that with *CAES *and *CES*. Performance of the test was examined via simulations as well as analyses on data of rheumatoid arthritis and heroin addiction, which maintains nominal level under null hypothesis and showed comparable performance with permutation test.

**Conclusions:**

*PCA-BCIT *is a valid and powerful method for assessing gene-disease association involving multiple SNPs.

## Background

Genetic association studies now customarily involve multiple SNPs in candidate genes or genomic regions and have a significant role in identifying and characterizing disease-predisposing variant(s). A critical challenge in their statistical analysis is how to make optimal use of all available information. Population-based case-control studies have been very popular[1] and typically involve contingency table tests of SNP-disease association[2]. Notably, the genotype-wise Armitage trend test does not require *HWE *and has equivalent power to its allele-wise counterpart under *HWE*[3,4]. A thorny issue with individual tests of SNPs for linkage disequilibrium (*LD*) in such setting is multiple testing, however, methods for multiple testing adjustment assuming independence such as Bonferroni's[5,6] is knowingly conservative[7]. It is therefore necessary to seek alternative approaches which can utilize multiple SNPs simultaneously. The genotype-wise Armitage trend test is appealing since it is equivalent to the score test from logistic regression[8] of case-control status on dosage of disease-predisposing alleles of SNP. However, testing for the effects of multiple SNPs simultaneously via logistic regression is no cure for difficulty with multicollinearity and curse of dimensionality[9]. Haplotype-based methods have many desirable properties[10] and could possibly alleviate the problem[11-14], but assumption of *HWE *is usually required and a potentially large number of degrees of freedom are involved[7,11,15-18].

It has recently been proposed that *PCA *can be combined with logistic regression test (*LRT*)[7,16,17] in a unified framework so that *PCA *is conducted first to account for between-SNP correlations in a candidate region, then *LRT *is applied as a formal test for the association between *PC *scores (linear combinations of the original SNPs) and disease. Since *PC*s are orthogonal, it avoids multicollinearity and at the meantime is less computer-intensive than haplotype-based methods. Studies have shown that *PCA-LRT *is at least as powerful as genotype- and haplotype-based methods[7,16,17]. Nevertheless, the power of *PCA*-based approaches vary with ways by which *PC*s are extracted, e.g., from genotype correlation, LD, or other kinds of metrics[17], and in principle can be employed in frameworks other than logistic regression[7,16,17]. Here we investigate ways of extracting *PCs *using genotype correlation matrix from different types of samples in a case-control study, while presenting a new approach testing for gene-disease association by direct use of *PC *scores in a *PCA*-based bootstrap confidence interval test (*PCA-BCIT*). We evaluated its performance via simulations and compared it with *PCA-LRT *and permutation test using real data.

## Methods

### PCA

Assume that *p *SNPs in a candidate region of interest have coded values (*X*_1_, *X*_2_, ⋯, *X*_*p*_) according to a given genetic model (e.g., additive model) whose correlation matrix is *C*. *PCA *solves the following equation,(1)

where  = 1, *i *= 1,2, ⋯, *p*, *l*_*i *_= (*l*_*i*1_, *l*_*i*2_, ⋯, *l*_*ip*_)' are loadings of *PC*s. The score for an individual subject is(2)

where cov (*F*_*i*_, *F*_*j*_) = 0, *i *≠ *j*, and var(*F*_1_) ≥ var(*F*_2_) ≥ ⋯ ≥ var(*F*_*p*_).

### Methods of extracting *PC*s

Potentially, *PCA *can be conducted via four distinct extracting strategies (*ES*) using case-control data, i.e., 0. Calculate *PC *scores of individuals in cases and controls separately (*SES*), 1. Use cases only (*CAES*) to obtain loadings for calculation of *PC *scores for subjects in both cases and controls, 2. Use controls only (*COES*) to obtain the loadings for both groups, and 3. Use combined cases and controls (*CES*) to obtain the loadings for both groups. It is likely that in a case-control association study, loadings calculated from cases and controls can have different connotations and hence we only consider scenarios 1-3 hereafter. More formally, let (*X*_1_, *X*_2_, ⋯, *X*_*p*_) and (*Y*_1_, *Y*_2_, ⋯, *Y*_*p*_) be *p*-dimension vectors of SNPs at a given candidate region for cases and controls respectively, then we have,

Strategy 1 (***CAES***):(3)

where *C*_*XX *_is the correlation matrix of (*X*_1_, *X*_2_, ⋯, *X*_*p*_),  and  = 1, *i *= 1,2, ⋯, *p*. The *i*^*th *^*PC *for cases is calculated by(4)

and for controls(5)

Strategy 2 (***COES***):(6)

where *C*_*YY *_is the correlation matrix of (*Y*_1_, *Y*_2_, ⋯, *Y*_*p*_). The *i*^*th *^*PC *for controls is calculated by(7)

And for cases, the *i*^*th *^*PC*, i = 1,2, ⋯, *p*, is calculated by(8)

Strategy 3 (***CES***):(9)

where *C *is the correlation matrix obtained from the pooled data of cases and controls,  and . The *i*^*th *^*PC *of cases is calculated by(10)

The *i*^*th *^*PC *of controls is calculated by(11)

### PCA-BCIT

Given a sample of *N *cases and *M *controls with *p*-SNP genotypes (*X*_1_, *X*_2_, ⋯, *X*_*N*_)^*T*^, (*Y*_1_, *Y*_2_, ⋯, *Y*_*M*_)^*T*^, and *X*_*i *_= (*X*_1*i*_, *X*_2*i*_, ⋯, *x*_*pi*_) for the *i*^*th *^case, *Y*_*i *_= (*Y*_1*i*_, *Y*_2*i*_, ⋯, *y*_*pi*_) for the *i*^*th *^control, a *PCA-BCIT *is furnished in three steps:

#### Step 1: Sampling

Replicate samples of cases and controls are obtained with replacement separately from (*X*_1_^(*b*^, *X*_2_^(*b*)^, ⋯, *X*_*N*_^(*b*)^)^*T *^and (*Y*_1_^(*b*^, *Y*_2_^(*b*)^, ⋯, *Y*_*M*_^(*b*)^)^*T*^, *b *= 1,2, ⋯, *B *(*B *= 1000).

#### Step 2: *PCA*

For each replicate sample obtained at Step 1, *PCA *is conducted and a given number of *PC*s retained with a threshold of 80% explained variance for all three strategies[16], expressed as  and .

#### Step 3: *PCA-BCIT*

**3a**) For each replicate, the mean of the *k*^*th *^*PC *in cases is calculated by(12)

and that of the *k*^*th *^*PC *in controls is calculated by(13)

**3b**) Given confidence level (1 - α ), the confidence interval of  is estimated by percentile method, with form(14)

where  is the  percentile of , and  is the  percentile.

The confidence interval of  is estimated by(15)

where  is the  percentile of , and  is the  percentile.

**3c**) Confidence intervals of cases and controls are compared. The null hypothesis is rejected if  and  do not overlap, which is  and  are statistically different[19], indicating the candidate region is significantly associated with disease at level α. Otherwise, the candidate region is not significantly associated with disease at level α.

### Simulation studies

**Figure 1:**
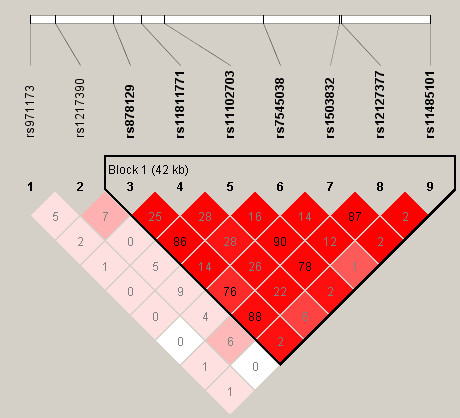
**LD (*r*^2^) among nine *PTPN22 *SNPs**. The nine *PTPN22 *SNPs are rs971173, rs1217390, rs878129, rs11811771, rs11102703, rs7545038, rs1503832, rs12127377, rs11485101. The triangle marks a single LD block within this region: (rs878129, rs11811771, rs11102703, rs7545038, rs1503832, rs12127377, rs11485101).

We examine the performance of *PCA-BCIT *through simulations with data from the North American Rheumatoid Arthritis (RA) Consortium (NARAC) (868 cases and 1194 controls)[20], taking advantage of the fact that association between protein tyrosine phosphatase non-receptor type 22 (*PTPN22*) and the development of RA has been established[21-24]. Nine SNPs have been selected from the *PNPT22 *region (114157960-114215857), and most of the SNPs are within the same LD block (Figure 1). Females are more predisposed (73.85%) and are used in our simulation to ensure homogeneity. The corresponding steps for the simulation are as follows.

#### Step 1: Sampling

The observed genotype frequencies in the study sample are taken to be their true frequencies in populations of infinite sizes. Replicate samples of cases and controls of given size (*N*, *N *= 100, 200, ⋯, 1000) are generated whose estimated genotype frequencies are expected to be close to the true population frequencies while both the allele frequencies and *LD *structure are maintained. Under null hypothesis, replicate cases and controls are sampled with replacement from the controls. Under alternative hypothesis, replicate cases and controls are sampled with replacement from the cases and controls respectively.

#### Step 2: *PCA-BCITing*

For each replicate sample, *PCA-BCITs *are conducted through the three strategies of extracting *PC*s as outlined above on association between *PC *scores and disease (RA).

#### Step 3: Evaluating performance of *PCA-BCIT*s

Repeat steps 1 and 2 for *K *( *K *= 1000 ) times under both null and alternative hypotheses, and obtain the frequencies (*P*_*α*_) of rejecting null hypothesis at level α (α = 0.05).

### Applications

**Figure 2:**
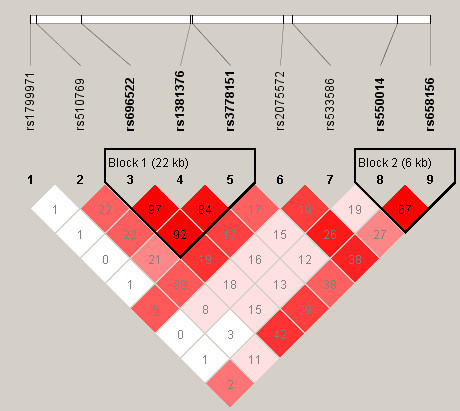
**LD (*r*^2^) among nine *OPRM1 *SNPs**. The nine *OPRM1 *SNPs are rs1799971, rs510769, rs696522, rs1381376, rs3778151, rs2075572, rs533586, rs550014, rs658156. The triangles mark the LD block 1 (rs696522, rs1381376, rs3778151) and LD block 2 (rs550014, rs658156).

*PCA-BCITs *are applied to both the NARAC data on *PTPN22 *in 1493 females (641 cases and 852 controls) described above and a data containing nine SNPs near μ-opioid receptor gene (*OPRM1*) in Han Chinese from Shanghai (91 cases and 245 controls) with endophenotype of heroin-induced positive responses on first use[25]. There are two LD blocks in the region of gene *OPRM1 *(Figure 2).

## Results

### Simulation study

The performance of *PCA-BCIT *is shown in Table 1 for the three strategies given a range of sample sizes. It can be seen that strategies 2 and 3 both have type I error rates approaching the nominal level (α = 0.05), but those from strategy 1 deviate heavily. When sample size larger than 800, the power of *PCA-BCIT *is above 0.8, and strategies 2 and 3 outperform strategy 1 slightly.

**Table 1:** Performance of *PCA-BCIT *at level 0.05 with strategies 1-3†

Sample size	Type I error			Power		
	
	*1*	*2*	*3*	*1*	*2*	*3*
100	0.014	0.036	0.037	0.156	0.163	0.176
200	0.016	0.044	0.036	0.249	0.278	0.292
300	0.017	0.028	0.029	0.383	0.426	0.368
400	0.014	0.04	0.02	0.508	0.485	0.516
500	0.009	0.035	0.042	0.613	0.595	0.597
600	0.006	0.032	0.042	0.677	0.662	0.683
700	0.007	0.061	0.04	0.733	0.758	0.73
800	0.004	0.043	0.045	0.801	0.791	0.819
900	0.005	0.057	0.051	0.826	0.855	0.858
1000	0.01	0.056	0.05	0.871	0.901	0.889

**†**1 case-only extracting strategy (*CAES*), 2 control-only extracting strategy (*COES*), 3 case-control extracting strategy (*CES*)

### Applications

For the NARAC data, Armitage trend test reveals none of the SNPs in significant association with RA using Bonferroni correction (Table 2), but the results of *PCA-BCIT *with strategies 2 and 3 show that the first *PC *extracted in region of *PTPN22 *is significantly associated with RA. The results are similar to that from permutation test (Table 3).

**Table 2:** Armitage trend test on nine *PTPN2*2 SNPs and RA susceptibility

SNP	Genotype	Female			Male		
		
		Case	Control	*P*-value	Case	control	*P*-value
rs971173	CC	334	381	0.025	116	169	0.779
	AC	236	363		85	134	
	AA	71	106		26	39	
rs1217390	AA	268	319	0.333	99	112	0.108
	AG	272	392		89	175	
	GG	98	138		38	55	
rs878129	GG	338	507	0.009	131	187	0.384
	AG	251	291		83	130	
	AA	52	54		13	25	
rs11811771	AA	224	272	0.090	78	111	0.717
	AG	303	411		104	168	
	GG	112	169		45	62	
rs11102703	CC	312	469	0.024	121	174	0.418
	AC	269	314		90	137	
	AA	60	69		16	31	
rs7545038	GG	321	428	0.696	109	186	0.417
	AG	265	342		98	114	
	AA	52	80		20	40	
rs1503832	AA	324	487	0.013	129	185	0.249
	AG	262	306		86	127	
	GG	55	59		12	30	
rs12127377	AA	349	521	0.017	139	197	0.230
	AG	243	282		78	121	
	GG	49	48		10	24	
rs11485101	AA	564	738	0.656	206	305	0.430
	AG	72	112		21	35	
	GG	5	2		0	2	

None of the P-values is significant after Bonferroni Correction.

**Table 3:** *PCA-BCIT*, *PCA-LRT *and permutation test on real data

Study	Strategy†	*99%CI*	*95%CI*	*P*-value‡	
				
				*PCA-LRT*	Permutation test
*PTPN22*	*2*	(-5.4E-01,-4.7E-03)** (-7.5E-16,6.9E-16)	(-4.8E-01,-8.6E-02)* (-4.6E-16,4.2E-16)	0.006**	0.002**
	*3*	(1.7E-02,3.3E-01)** (-2.5E-01,-1.3E-02)	(4.9E-02,3.0E-01)* (-2.2E-01,-3.7E-02)	0.007**	0.002**
*OPRM1*	*2*	(-1.2E+00,-1.1E-02)** (-4.7E-16,5.0E-16)	(-1.1E+00,-1.8E-01)* (-3.7E-16,3.4E-16)	0.107	0.002**
	*3*	(5.3E-02,1.4E+00)** (-4.9E-01,-1.7E-02)	(2.4E-01,1.2E+00)* (-4.2E-01,-8.0E-02)	0.012*	0.004**

**†**2 control-only extracting strategy (*COES*), 3 case-control extracting strategy (*CES*)

‡* significant at levels α = 0.05(*) and α = 0.01 (**).

**Figure 3:**
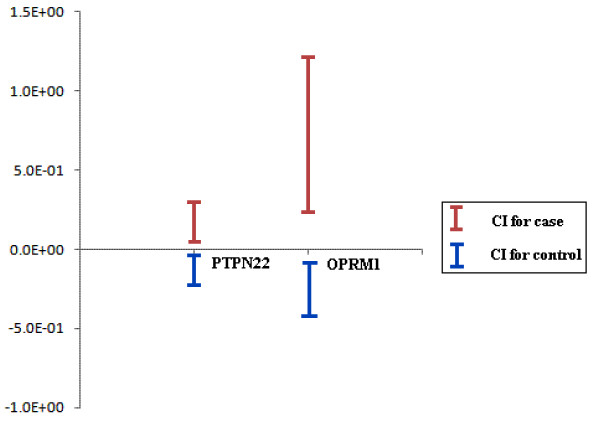
**Real data analyses by *PCA-BCIT *with strategy 3 and confidence level 0.95**. The horizontal axis denotes studies and vertical axis *mean(PC1)*, the statistic used to calculate confidence intervals for cases and controls. *PCA-BCIT*s with strategy 3 were significant at confidence level 0.95.

For the *OPRM1 *data, the sample characteristics are comparable between cases and controls (Table 4), and three SNPs (rs696522, rs1381376 and rs3778151) are showed significant association with the endophenotype (Table 5). The results of *PCA-BCIT *with strategies 2 and 3 and permutation test are all significant at level α = 0.01. In contrast, result from *PCA-LRT *is not significant at level α = 0.05 with strategy 2 (Table 3). The apparent separation of cases and controls are shown in Figure 3 for *PCA-BCIT *with strategy 3, suggesting an intuitive interpretation.

**Table 4:** Sample characteristics of heroin-induced positive responses on first use

	Cases (*N *= 91)	Controls (*N *= 245)	*P*-value
Age (yrs)	30.42 ± 7.65	30.93 ± 8.18	0.6057
Women (%)	26.4	29.8	0.5384
Age at onset (yrs)	26.29 ± 7.41	26.97 ± 7.89	0.4760
Reason for first use of heroin			0.7173
Curiousness	79.1	75.1	
Peer pressure	6.6	4.9	
Physical disease	7.7	10.2	
Trouble	5.5	6.1	
Other reasons	1.1	3.8	

**Table 5:** Armitage trend tests on nine *OPRM1 *SNPs and heroin-induced positive responses on first use

SNP	Genotype	Count and frequency				Armitage trend test	
		
		Cases		Controls		Chi-square	*P*-value
rs1799971	AA	55	0.604	150	0.622	0.003	0.9537
	AG	27	0.297	64	0.266		
	GG	9	0.099	24	0.112		
rs510769	TT	56	0.667	167	0.749	2.744	0.0976
	TC	24	0.286	53	0.237		
	CC	4	0.048	4	0.018		
rs696522	AA	64	0.762	215	0.907	11.097	0.0009*
	AG	19	0.226	21	0.089		
	GG	1	0.012	1	0.004		
rs1381376	CC	70	0.769	221	0.913	13.409	0.0003*
	CT	20	0.220	21	0.087		
	TT	1	0.011	0	0.000		
rs3778151	GG	66	0.733	215	0.896	14.655	0.0001*
	GA	23	0.256	25	0.104		
	AA	1	0.011	0	0.000		
rs2075572	GG	50	0.556	149	0.642	1.574	0.2096
	GC	33	0.367	82	0.353		
	CC	7	0.078	11	0.047		
rs533586	TT	68	0.840	203	0.868	0.761	0.3830
	TC	12	0.148	31	0.132		
	CC	1	0.012	0	0.000		
rs550014	TT	78	0.857	203	0.832	0.093	0.7602
	TC	12	0.132	41	0.168		
	CC	1	0.011	0	0.000		
rs658156	GG	65	0.714	192	0.787	2.041	0.1531
	GA	24	0.264	52	0.213		
	AA	1	0.011	0	0.000		

* significant after Bonferroni Correction.

## Discussion

In this study, a *PCA*-based bootstrap confidence interval test[19,26-28] (*PCA*-*BCIT*) is developed to study gene-disease association using all SNPs genotyped in a given region. There are several attractive features of *PCA*-based approaches. First of all, they are at least as powerful as genotype- and haplotype-based methods[7,16,17]. Secondly, they are able to capture LD information between correlated SNPs and easy to compute with needless consideration of multicollinearity and multiple testing. Thirdly, *BCIT *integrates point estimation and hypothesis testing as a single inferential statement of great intuitive appeal[29] and does not rely on the distributional assumption of the statistic used to calculate confidence interval[19,26-29].

While there have been several different but closely related forms of bootstrap confidence interval calculations[28], we focus on percentiles of the asymptotic distribution of *PC*s for given confidence levels to estimate the confidence interval. *PCA-BCIT *is a data-learning method[29], and shown to be valid and powerful for sufficiently large number of replicates in our study. Our investigation involving three strategies of extracting *PC*s reveals that strategy 1 is invalid, while strategies 2 and 3 are acceptable. From analyses of real data we find that *PCA-BCIT *is more favourable compared with *PCA-LRT *and permutation test. It is suggested that a practical advantage of *PCA-BCIT *is that it offers an intuitive measure of difference between cases and controls by using the set of SNPs (*PC *scores) in a candidate region (Figure 3). As extraction of *PC*s through *COES *is more in line with the principle of a case-control study, it will be our method of choice given that it has a comparable performance with *CES*. Nevertheless, *PCA-BCIT *has the limitation that it does not directly handle covariates as is usually done in a regression model.

## Conclusions

*PCA-BCIT *is both a valid and a powerful *PCA*-based method which captures multi-SNP information in study of gene-disease association. While extracting *PC*s based on *CAES, COES *and *CES *all have good performances, it appears that *COES *is more appropriate to use.

## Abbreviations

*SNP*: single nucleotide polymorphism; *HWE*: Hardy-Weinberg Equilibrium; *LD*: linkage disequilibrium; *LRT*: logistic regression test; *PCA*: principle component analysis; *PC*: principle component; *ES*: extracting strategy; *SES*: separate case and control extracting strategy (strategy 0); *CAES*: case-based extracting strategy (strategy 1); *COES*: control-based extracting strategy (strategy 2); *CES*: combined case and control extracting strategy (strategy 3); *BCIT*: bootstrap confidence interval test.

## Authors' contributions

QQP, JHZ, and FZX conceptualized the study, acquired and analyzed the data and prepared for the manuscript. All authors approved the final manuscript.

## References

[B1] Morton NE Collins A Tests and estimates of allelic association in comples Proc Natl Acad Sci USA 1998 95 11389 11393 10.1073/pnas.95.19.11389 9736746 PMC21652

[B2] Sasieni PD From genotypes to genes: doubling the sample size Biometrics 1997 53 1253 1261 10.2307/2533494 9423247

[B3] Gordon D Haynes C Yang Y Kramer PL Finch SJ Linear trend tests for case-control genetic association that incorporate random phenotype and genotype misclassification error Genet Epidemiol 2007 31 853 870 10.1002/gepi.20246 17565750

[B4] Slager SL Schaid DJ Case-control studies of genetic markers: Power and sample size approximations for Armitage's test for trend Human Heredity 2001 52 149 153 10.1159/000053370 11588398

[B5] Sidak Z On Multivariate Normal Probabilities of Rectangles: Their Dependence on Correlations The Annals of Mathematical Statistics 1968 39 1425 1434

[B6] Sidak Z On Probabilities of Rectangles in Multivariate Student Distributions: Their Dependence on Correlations The Annals of Mathematical Statistics 1971 42 169 175 10.1214/aoms/1177693504

[B7] Zhang FY Wagener D An approach to incorporate linkage disequilibrium structure into genomic association analysis Journal of Genetics and Genomics 2008 35 381 385 10.1016/S1673-8527(08)60055-7 18571127 PMC2746675

[B8] Balding DJ A tutorial on statistical methods for population association studies Nature Reviews Genetics 2006 7 781 791 10.1038/nrg1916 16983374

[B9] Schaid DJ McDonnell SK Hebbring SJ Cunningham JM Thibodeau SN Nonparametric tests of association of multiple genes with human disease American Journal of Human Genetics 2005 76 780 793 10.1086/429838 15786018 PMC1199368

[B10] Becker T Schumacher J Cichon S Baur MP Knapp M Haplotype interaction analysis of unlinked regions Genetic Epidemiology 2005 29 313 322 10.1002/gepi.20096 16240441

[B11] Chapman JM Cooper JD Todd JA Clayton DG Detecting disease associations due to linkage disequilibrium using haplotype tags: A class of tests and the determinants of statistical power Human Heredity 2003 56 18 31 10.1159/000073729 14614235

[B12] Epstein MP Satten GA Inference on haplotype effects in case-control studies using unphased genotype data American Journal of Human Genetics 2003 73 1316 1329 10.1086/380204 14631556 PMC1180397

[B13] Fallin D Cohen A Essioux L Chumakov I Blumenfeld M Cohen D Schork NJ Genetic analysis of case/control data using estimated haplotype frequencies: Application to APOE locus variation and Alzheimer's disease Genome Research 2001 11 143 151 10.1101/gr.148401 11156623 PMC311030

[B14] Stram DO Pearce CL Bretsky P Freedman M Hirschhorn JN Altshuler D Kolonel LN Henderson BE Thomas DC Modeling and E-M estimation of haplotype-specific relative risks from genotype data for a case-control study of unrelated individuals Human Heredity 2003 55 179 190 10.1159/000073202 14566096

[B15] Clayton D Chapman J Cooper J Use of unphased multilocus genotype data in indirect association studies Genetic Epidemiology 2004 27 415 428 10.1002/gepi.20032 15481099

[B16] Gauderman WJ Murcray C Gilliland F Conti DV Testing association between disease and multiple SNPs in a candidate gene Genetic Epidemiology 2007 31 383 395 10.1002/gepi.20219 17410554

[B17] Oh S Park T Association tests based on the principal-component analysis BMC Proc 2007 1 Suppl 1 S130 10.1186/1753-6561-1-s1-s130 18466473 PMC2367557

[B18] Wang T Elston RC Improved power by use of a weighted score test for linkage disequilibrium mapping American Journal of Human Genetics 2007 80 353 360 10.1086/511312 17236140 PMC1785334

[B19] Heller G Venkatraman ES Resampling procedures to compare two survival distributions in the presence of right-censored data Biometrics 1996 52 1204 1213 10.2307/2532836

[B20] Plenge RM Seielstad M Padyukov L Lee AT Remmers EF Ding B Liew A Khalili H Chandrasekaran A Davies LRL TRAF1-C5 as a risk locus for rheumatoid arthritis - A genomewide study New England Journal of Medicine 2007 357 1199 1209 10.1056/NEJMoa073491 17804836 PMC2636867

[B21] Begovich AB Carlton VE Honigberg LA Schrodi SJ Chokkalingam AP Alexander HC Ardlie KG Huang Q Smith AM Spoerke JM A missense single-nucleotide polymorphism in a gene encoding a protein tyrosine phosphatase (PTPN22) is associated with rheumatoid arthritis Am J Hum Genet 2004 75 330 337 10.1086/422827 15208781 PMC1216068

[B22] Carlton VEH Hu XL Chokkalingam AP Schrodi SJ Brandon R Alexander HC Chang M Catanese JJ Leong DU Ardlie KG PTPN22 genetic variation: Evidence for multiple variants associated with rheumatoid arthritis American Journal of Human Genetics 2005 77 567 581 10.1086/468189 16175503 PMC1275606

[B23] Kallberg H Padyukov L Plenge RM Ronnelid J Gregersen PK Helm-van Mil AHM van der Toes REM Huizinga TW Klareskog L Alfredsson L Gene-gene and gene-environment interactions involving HLA-DRB1, PTPN22, and smoking in two subsets of rheumatoid arthritis American Journal of Human Genetics 2007 80 867 875 10.1086/516736 17436241 PMC1852748

[B24] Plenge RM Padyukov L Remmers EF Purcell S Lee AT Karlson EW Wolfe F Kastner DL Alfredsson L Altshuler D Replication of putative candidate-gene associations with rheumatoid arthritis in > 4,000 samples from North America and Sweden: Association of susceptibility with PTPN22, CTLA4, and PADI4 American Journal of Human Genetics 2005 77 1044 1060 10.1086/498651 16380915 PMC1285162

[B25] Zhang D Shao C Shao M Yan P Wang Y Liu Y Liu W Lin T Xie Y Zhao Y Effect of mu-opioid receptor gene polymorphisms on heroin-induced subjective responses in a Chinese population Biol Psychiatry 2007 61 1244 1251 10.1016/j.biopsych.2006.07.012 17157823

[B26] Carpenter J Test Inversion Bootstrap Confidence Intervals Journal of the Royal Statistical Society Series B (Statistical Methodology) 1999 61 159 172 10.1111/1467-9868.00169

[B27] Davison AC Hinkley DV Young GA Recent developments in bootstrap methodology Statistical Science 2003 18 141 157 10.1214/ss/1063994969

[B28] DiCiccio TJ Efron B Bootstrap confidence intervals Statistical Science 1996 11 189 212 10.1214/ss/1032280214

[B29] Efron B Bootstrap Methods: Another Look at the Jackknife The Annals of Statistics 1979 7 1 26 10.1214/aos/1176344552

